# Reliability and validity of PRO-CTCAE® daily reporting with a 24-hour recall period

**DOI:** 10.1007/s11136-023-03374-5

**Published:** 2023-03-10

**Authors:** M. K. Lee, E. Basch, S. A. Mitchell, L. M. Minasian, B. T. Langlais, G. Thanarajasingam, B. F. Ginos, L. J. Rogak, T. R. Mendoza, A. V. Bennett, D. Schrag, G. L. Mazza, A. C. Dueck

**Affiliations:** 1 Department of Quantitative Health Sciences, Mayo Clinic, Rochester, MN USA; 2 UNC Lineberger Comprehensive Cancer Center, Chapel Hill, NC USA; 3 National Cancer Institute, Rockville, MD USA; 4 Department of Quantitative Health Sciences, Mayo Clinic, Scottsdale, AZ USA; 5 Division of Hematology, Mayo Clinic, Rochester, MN USA; 6 Memorial Sloan Kettering Cancer Center, New York, NY USA; 7 MD Anderson Cancer Center, Houston, TX USA

**Keywords:** PRO-CTCAE, Daily diary, 24-hour recall, Reliability, Validity

## Abstract

**Purpose:**

The standard recall period for the patient-reported outcomes version of the common terminology criteria for adverse events (PRO-CTCAE®) is the past 7 days, but there are contexts where a 24-hour recall may be desirable. The purpose of this analysis was to investigate the reliability and validity of a subset of PRO-CTCAE items captured using a 24-hour recall.

**Methods:**

27 PRO-CTCAE items representing 14 symptomatic adverse events (AEs) were collected using both a 24-hour recall (24 h) and the standard 7 day recall (7d) in a sample of patients receiving active cancer treatment (*n* = 113). Using data captured with a PRO-CTCAE-24h on days 6 and 7, and 20 and 21, we computed intra-class correlation coefficients (ICC); an ICC ≥ 0.70 was interpreted as demonstrating high test–retest reliability. Correlations between PRO-CTCAE-24h items on day 7 and conceptually relevant EORTC QLQ-C30 domains were examined. In responsiveness analysis, patients were deemed changed if they had a one-point or greater change in the corresponding PRO-CTCAE-7d item (from week 0 to week 1).

**Results:**

PRO-CTCAE-24h captured on two consecutive days demonstrated that 21 of 27 items (78%) had ICCs ≥ 0.70 (day 6/7 median ICC 0.76), (day 20/21 median ICC 0.84). Median correlation between attributes within a common AE was 0.75, and the median correlation between conceptually relevant EORTC QLQ-C30 domains and PRO-CTCAE-24 h items captured on day 7 was 0.44. In the analysis of responsiveness to change, the median standardized response mean (SRM) for patients with improvement was − 0.52 and that for patients with worsening was 0.71.

**Conclusion:**

A 24-hour recall period for PRO-CTCAE items has acceptable measurement properties and can inform day-to-day variations in symptomatic AEs when daily PRO-CTCAE administration is implemented in a clinical trial.

**Supplementary Information:**

The online version contains supplementary material available at 10.1007/s11136-023-03374-5.

## Plain English summary

The purpose of this study was to evaluate if a shorter recall period for the patient-reported outcomes version of the common terminology criteria for adverse events (PRO-CTCAE) was reliable and valid for the intended population. This analysis used existing data from a study where a small number of patients were asked to answer 27 PRO-CTCAE items representing 14 symptomatic adverse events (AEs) using a 24-hour recall in addition to the standard 7-day recall period. In this analysis, we examined test–retest reliability for two consecutive days of reporting with a 24-hour recall. Validity was evaluated by correlations among PRO-CTCAE items, as well as correlations of PRO-CTCAE items with EORTC QLQ-C30 scales, and through responsiveness to change analyses. This study showed that the 27 PRO-CTCAE items with a 24-hour recall period had mostly acceptable measurement properties and when PRO-CTCAE is administered daily, can be used to capture day-to-day variations in symptomatic AEs, although more work needs to be done.

## Introduction

Patient-reported outcomes can provide clinically relevant information on symptomatic adverse events (AEs) as part of the assessment of cancer treatment tolerability [1, 2]. The US National Cancer Institute (NCI)’s patient-reported outcomes version of the common terminology criteria for adverse events (PRO-CTCAE®) is an item library designed to capture symptomatic adverse events in cancer clinical trials. It has been developed using rigorous qualitative and quantitative methods and demonstrates favorable measurement properties, including reliability, validity, responsiveness, and mode equivalence [1, 3–5]. In any given cancer clinical trial, investigators select a subset of relevant items from this library. The current evidence base supports the use of PRO-CTCAE to provide descriptive reporting of symptomatic toxicities along with the CTCAE grading by clinicians, and PRO-CTCAE is currently in use in many global cancer trials [6].

The standard recall period for the PRO-CTCAE is ‘the last 7 days.’ Mendoza et al. [7] have shown that the 7-day recall period is preferable to longer recall periods due to the maximum amount of information retention, and 2-week and 3-week recall periods also perform well and may be appropriate in specific research or clinical contexts. However, a shorter recall period and daily reporting may be warranted when detailed recall of experience is needed to address a specific research question or when symptoms may be anticipated to fluctuate from day to day. The measurement properties of PRO-CTCAE when responses are captured using a 24-hour recall period are unknown; such information would permit investigators to select the recall period that best aligns with their research objectives and the schedule of assessments.

The US Food and Drug Administration (FDA) 2009 guidance for industry on patient-reported outcome (PRO) measures [8] recommended key psychometric properties, including (1) test–retest reliability which is the ability to yield consistent, reproducible estimates of true scores, (2) construct validation as is evidenced by logical relationships that should exist between the new measure and other related measures, and (3) responsiveness to change, which is the ability to detect change when there has been meaningful change, a concept akin to criterion validation. While the ‘last 7 days’ is the standard recall period for the PRO-CTCAE measurement system, a shorter recall period of the ‘last 24 h’ could allow for more flexibility in study contexts where daily reporting of symptomatic adverse events is warranted. Therefore, the purpose of this analysis was to investigate the reliability and validity of daily reporting of PRO-CTCAE items using a 24-hour recall period.

## Methods

### Settings and samples

This analysis utilized a subset of the data collected previously as part of a PRO-CTCAE validation study [5] conducted in collaboration with the US National Cancer Institute (clinicaltrials.gov NCT02158637). This study enrolled US-residing, English-speaking adult patients with a solid tumor or hematologic malignancy who were scheduled to receive cancer treatment within the next 7 days, and who were without clinically significant cognitive impairment. Four US-based cancer centers participated in this study (Dana-Farber Cancer Institute, Boston, MA; Mayo Clinic, Rochester, MN; Memorial Sloan Kettering Cancer Center, New York, NY; The University of Texas MD Anderson Cancer Center, Houston, TX). Institutional review board approval was obtained from each site and at the National Cancer Institute. Eligible patients were approached in clinic waiting areas to participate, and their written informed consent was obtained. Eligible participants had (i) metastatic or locally advanced lung cancer or head/neck/gastroesophageal cancer and were receiving daily radiation therapy (± concurrent chemotherapy) for at least 21 days; or (ii) breast, lymphoma, myeloma, prostate, bladder, lung, or colorectal cancer who were starting chemotherapy within the next 7 days or were currently receiving chemotherapy and projected to visit the clinic for at least 4–5 consecutive weekly clinic visits.

### Measures

#### PRO-CTCAE

Twenty seven PRO-CTCAE items were administered each day using a 24-hour recall in the parent substudy [7]. These items reflected 14 common and cross-cutting symptomatic adverse events [9] and included anxiety, sad or unhappy feelings, constipation, loose stools, loss of appetite, nausea, shortness of breath, numbness or tingling in hands and feet, pain, fatigue, insomnia, dry mouth, mouth or throat sores, and vomiting.

PRO-CTCAE items measure between 1 and 3 attributes of a given symptomatic AE, including frequency, severity, and/or interference with daily activities. Responses are captured with a Likert-type response scale ranging from never/none/not at all (numerical score 0) to almost constantly/very severe/very much (numerical score 4). Items that were conditionally skipped, were scored as 0. For the daily reporting, each item started with the modified recall period, e.g., “in the last 24 hours”; otherwise, the format, language and response options of the 24-hour recall items were identical to the 7-day recall items. Example items are shown in Supplemental Table 1.

#### EORTC QLQ-C30

The relationships between the PRO-CTCAE items collected on days 0 and 7 using a 24-hour recall and the European Organization for Research and Treatment of Cancer Core Quality of Life Questionnaire (EORTC QLQ-C30) were examined for evidence of convergent/discriminant validity as well as responsiveness to change [10]. The 30-item instrument produces an HRQOL summary score, a global health status/quality of life (QOL) scale score, five functioning scale scores, and eight symptom item/scale scores (excluding financial difficulties score). The EORTC QLQ-C30, which is based on a recall period of “the past week,” was administered via paper booklet at two time points, day 0 and day 7 (Table 1).

**Table 1:** The schedule of questionnaire administration

Week	0							1							2							3							4
Day	0	1	2	3	4	5	6	7	8	9	10	11	12	13	14	15	16	17	18	19	20	21	22	23	24	25	26	27	28
PRO-CTCAE 24-hour recall	X	X	X	X	X	X	X	X	X	X	X	X	X	X	X	X	X	X	X	X	X	X	X	X	X	X	X	X	X
PRO-CTCAE with a 7-day recall	X	X						X																					
PRO-CTCAE with a 2-week recall															X														
PRO-CTCAE with a 3-week recall																						X							
PRO-CTCAE with a 4-week recall																													X
EORTC QLQ-C30 weekly recall	X							X																					

A subset of 113 respondents completed 24-hour recall assessments out of 940 participants who completed PRO-CTCAE items in the larger validation study

### Study design

PRO-CTCAE items using a 24-hour recall (PRO-CTCAE-24 h) were administered daily via an automated telephone interactive voice response (IVR) system over a 4 week (28 day) period. Patients received IVR phone calls outside of clinic (i.e., on a home landline or personal cell phone). The PRO-CTCAE items using the standard 7-day recall (PRO-CTCAE-7d) were administered on day 0 (week 0) and day 7 (week 1) via the web during a scheduled clinic visit (Table 1). Therefore, the weekly report obtained at week 0 and week 1 asked patients to rate their symptoms during “the last 7 days.” For both 7-day and 24-hour recall periods, conditional branching was employed based on the first presented item only. For example, both the IVR system and web-based surveys implemented conditional branching where participants were not asked about an AE’s severity or interference if they reported not experiencing that AE (i.e., a frequency of “never”) [5].

### Statistical analyses

#### Test–retest reliability and intraindividual variability

Test–retest reliability is defined as the stability of scores over time when no change is expected in the construct of interest. We examined daily reports from two consecutive days (Days 6 and 7 and Days 20 and 21), based on the hypothesis that patients with stable symptoms would report similar scores from one day to the next. We used the intra-class correlation coefficient (ICC) formula recommended for test–retest reliability, which is based on the two-way mixed-effect analysis of variance model with time-by-subject interaction [11]. An ICC of 0.70 or greater was interpreted as high [12]. We additionally compared intraindividual variability on days 0 through 6 and then on days 15 through 21 by computing the within-individual coefficients of variation (CVI), the ratios of the standard deviation to the mean. We computed each individual’s CVI for each item and present the median and the interquartile range of the CVIs for each item.

#### Convergent and discriminant validity

We hypothesized that within-AE items should correlate highly with each other (i.e., convergent validation). Accordingly, we examined associations between PRO-CTCAE items measuring different attributes of a common AE (i.e., frequency, severity, interference). We hypothesized that items capturing related AEs (e.g., anxiety and feeling sad) would also correlate with each other, but the strength of that correlation would be lower than that observed between items evaluating the attributes of a common AE. Not only should items measuring common or similar constructs correlate with related items, but they should also not correlate with unrelated or dissimilar AEs (i.e., discriminant validation). We computed bivariate correlations using Spearman’s rank correlation coefficients (*ρ*). For this analysis, we used PRO-CTCAE-24 h that was captured on day 7 (Table 1). We used the 24-hour recall from day 7 rather than day 0 so that all participants would have commenced their treatment.

We also examined the associations between the EORTC QLQ-C30 scale scores and the day 7 PRO-CTCAE-24 h items using Spearman’s rank correlation coefficients. To aid interpretation, QLQ-C30 HRQOL summary and other functioning/symptom scale scores were reverse coded such that higher scores represent worse outcomes, matching the direction of PRO-CTCAE items.

#### Responsiveness to change

Responsiveness to change is the ability of an instrument to measure a meaningful change in a clinical state [13]. Responsiveness to change is an aspect of validity most akin to criterion validation [14] and may be gauged by examining whether the change detected by the new measure (PRO-CTCAE-24 h captured on day 0 and day 7) correlates with change as measured by another well-established instrument (i.e., PRO-CTCAE-7d captured on week 0 and week 1).

#### PRO-CTCAE with the 7 day recall period as an anchor

In the first set of analyses, the ability of the daily report to detect change was investigated by comparing the score change in PRO-CTCAE daily reporting (day 0 to day 7) to the score change in the PRO-CTCAE-7d (week 0 to week 1). Each PRO-CTCAE-7d change score for the same symptom and attribute (i.e., frequency, severity, interference) was used as the criterion for the corresponding change score in the PRO-CTCAE daily report. In addition, any one-point change in PRO-CTCAE-7d score from week 0 to week 1 was considered meaningful based on the study [15] that found each ordinal response choice in PRO-CTCAE served to distinguish respondents with meaningfully different symptom experiences. A one-point or greater worsening (e.g., score changes 3 to 4 from week 0 to week 1) in PRO-CTCAE-7d would classify a patient as ‘worse.’ Similarly, a one-point or greater improvement from week 0 to week 1 classified a respondent as ‘better.’ Unchanged scores from week 0 to week 1 were classified as ‘the same.’ Comparisons were made using a one-sided Jonckheere-Terpstra [16] test. This test evaluates whether the score distributions of a target measure have a monotonic ordering that parallels the ordered categories of a criterion measure. Change scores for each PRO-CTCAE-24 h (day 0 to 7) could theoretically range from − 4 to 4. We expected that the direction of the ordering of the PRO-CTCAE-24 h score distributions would parallel the scores changes in PRO-CTCAE-7d from week 0 to week 1, and thus, we used a single-tailed test. In addition, we calculated the standardized response mean (SRM; the ratio of the average change to the standard deviation of the change scores) as an index of responsiveness to change, for each of the 27 PRO-CTCAE-24 h daily reports by the change category informed by PRO-CTCAE-7d weekly report. Lastly, we examined the magnitude of the correlations between the change in PRO-CTCAE-24 h from day 0 and day 7, with changes in PRO-CTCAE-7d from week 0 to week 1.

#### EORTC QLQ-C30 as an anchor

In the second set of analyses, we correlated the changes in 15 PRO-CTCAE-24 h recall scores (day 0 to day 7) with changes in conceptually related EORTC QLQ-C30 item/scale scores (week 0 to week 1) via Spearman rank correlation coefficients. Specifically, correlations were investigated between EORTC QLQ-C30 fatigue scale score and PRO-CTCAE fatigue severity or fatigue interference items (S, I); correlations between EORTC QLQ-C30 nausea scale score was computed with PRO-CTCAE nausea frequency or nausea severity items (F, S); and correlations between EORTC QLQ-C30 item/scale scores and the related PRO-CTCAE counterparts were computed for vomiting (F, S), pain (F,S,I), dyspnea (S, I), insomnia (S), appetite (S, I), and constipation (S).

## Results

### Descriptive summaries of the sample and the PRO-CTCAE-24h items captured on day 7

There were 118 adult patients participating in this substudy who received either radiation therapy or chemotherapy or both within the two weeks prior to enrollment between January 2011 and February 2012 [7]. Of those 118, five individuals did not contribute data for any analyses. Thus, the analytic sample included 113 patients who contributed to one or more analyses. The sample had a median age of 58 (range, 20–77); 86% were White; 38% had lower educational attainment than a college degree. Participants were being treated for a variety of cancer sites, with an overrepresentation of lung and head and neck malignancies since a majority of the sample for this substudy was receiving daily radiation treatment. While the daily reporting occurred at home, daily treatment gave study participants an opportunity to seek staff assistance with the logistics of daily reporting if necessary. Our cohort was also assumed to experience fluctuating symptoms due to receiving daily treatment. Most participants (98%) had ECOG level of 0–1 (Table 2). However, the response rate dropped to 75% on day 1, and this level of response was generally maintained until day 21. By day 22, the response rate dropped to 65%, and by day 28, the response rate dropped further to 42%.

**Table 2:** Participant baseline characteristics

	Total (*N* = 113)	Those who provided PRO-CTCAE-24 h data on day 7 (*N* = 86)
Age at enrollment		
Mean (SD)	55.5 (12.50)	55.5 (12.76)
Median	57.0	57.0
Range	20.0, 77.0	20.0, 77.0
Age group, n (%)		
< 30	5 (4.4%)	4 (4.7%)
30–64	78 (69.0%)	56 (65.1%)
65–74	28 (24.8%)	25 (29.1%)
> = 75	2 (1.8%)	1 (1.2%)
Gender, n (%)		
Female	46 (40.7%)	33 (38.4%)
Male	67 (59.3%)	53 (61.6%)
Race, n (%)		
White	96 (85.0%)	72 (83.7%)
Black or African American	11 (9.7%)	8 (9.3%)
Asian	6 (5.3%)	6 (7.0%)
Ethnicity, n (%)		
Hispanic/Latino	6 (5.3%)	4 (4.7%)
Non-Hispanic	106 (93.8%)	82 (95.3%)
Unknown/Not reported	1 (0.9%)	0 (0%)
Education level, n (%)		
Less than high school	1 (0.9%)	1 (1.2%)
High school or GED	20 (17.7%)	15 (17.4%)
Some college	19 (16.8%)	16 (18.6%)
College graduate or more	70 (61.9%)	51 (59.3%)
Missing	3 (2.7%)	
Disease, n (%)		
Breast	11 (9.7%)	10 (11.6%)
Head/Neck	61 (54.0%)	46 (53.5%)
Lung	28 (24.8%)	19 (22.1%)
GI	5 (4.4%)	4 (4.7%)
Heme	8 (7.1%)	7 (8.1%)
ECOG PS (Visit 1), n (%)		
ECOG 0–1	110 (97.3%)	83 (96.5%)
ECOG 2–4	3 (2.7%)	3 (3.5%)

**Fig. 1:**
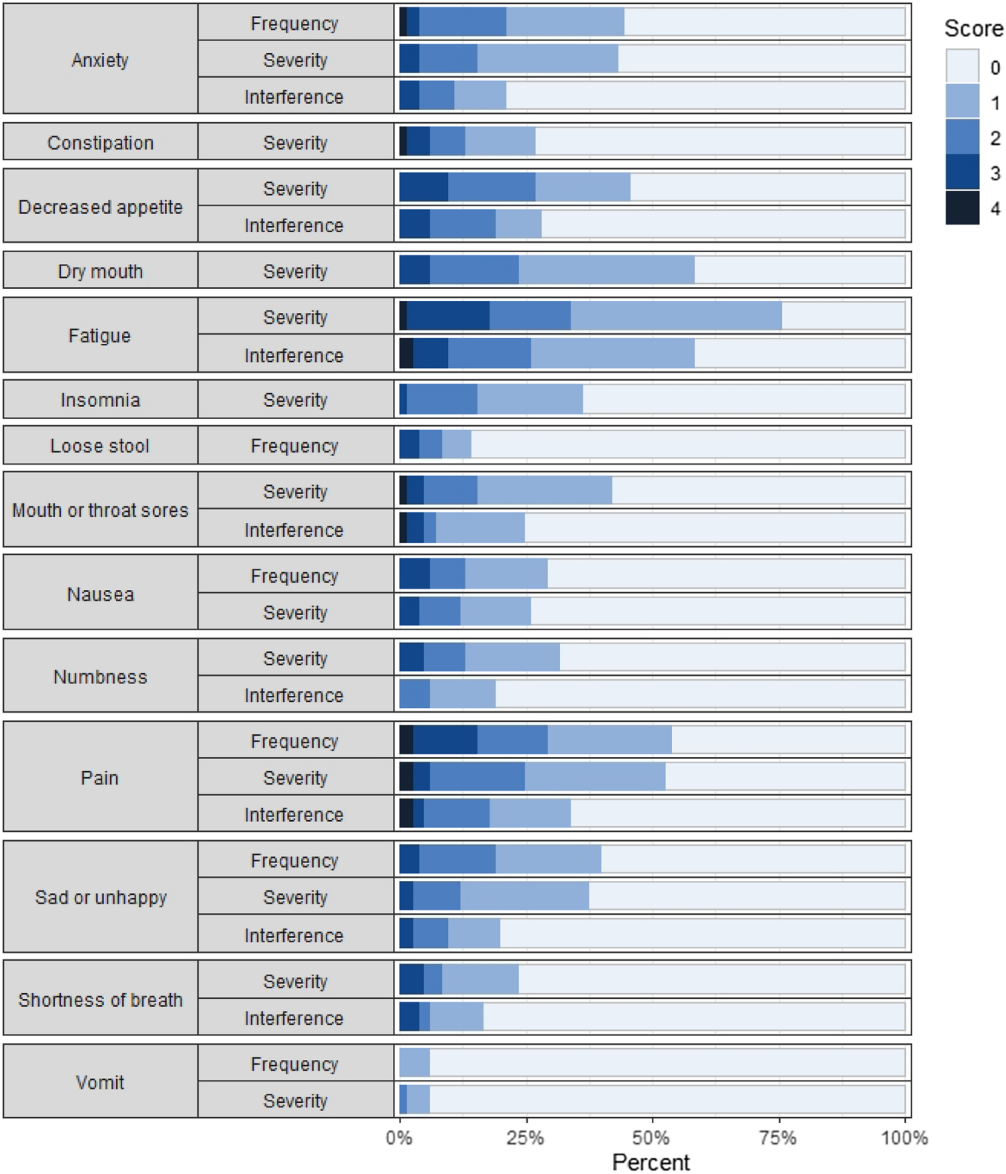
Stacked bar plots representing the percentages endorsing each response option for each of the 27 PRO-CTCAE items. (Day 7 24-hour recall, *N* = 86). There were 86 patients who responded to daily reports on day 7

Figure 1 displays stacked bar plots with percentages of participants (*n* = 86) endorsing each response option for each of the 27 items on day 7 using a 24-hour recall. The most commonly reported symptom was fatigue severity (77% prevalence). There were 7 patients who did not report the presence of any AEs. The number of items endorsed (i.e., response category of one or higher) per patient ranged from 0 to 24, and the median was 8. There were 6 items where the highest scoring response option (score 4) was not selected by respondents. These items were vomiting (S), vomiting (F), shortness of breath (S), sad or unhappy feelings (S), sad or unhappy feelings (F), and anxiety (S).

### Test–retest reliability and intraindividual variability

In 86 patients who completed the PRO-CTCAE-24 h on the 6th and 7th days of the study, the test–retest reliability estimates ranged from 0.36 to 0.90 (median ICC, 0.76), with 21 of 27 items having an ICC of at least 0.70 (Table 3). In 77 patients who completed the PRO-CTCAE-24 h on the 20th and 21st days of the trial, the test–retest reliability for the 27 items ranged from 0.51 to 0.97 (median ICC, 0.84) with 21 of 27 items having an ICC of at least 0.70. CVIs differed considerably depending upon the symptomatic AE, with symptoms such as sad or unhappy feelings (I), constipation (S), diarrhea (F), and vomiting (F, S) demonstrating considerable within-individual variability (i.e., CVI near or above 1), particularly from days 0 to 6. The CVIs became smaller on days 15–21 compared to days 0–6, indicating somewhat less within-subject variability in scores among the repeated measures captured on later dates (Supplemental Table 2).

**Table 3:** Test–retest reliability for 27 prespecified PRO-CTCAE-24 h items for two pairs of daily reports (Days 6 and 7, and days 20 and 21)

PRO-CTCAE symptomatic AE term	Intra-class correlation coefficient (95% C.I.)		
	Frequency	Severity	Interference
Day 6 and day 7 (24-hour recall) (*N* = 86)			
Anxiety	0.71 (0.58, 0.80)	0.72 (0.61, 0.81)	0.68 (0.55, 0.78)
Constipation		0.59 (0.44, 0.72)	
Decreased appetite		0.86 (0.79, 0.91)	0.72 (0.60, 0.81)
Dry mouth		0.85 (0.78, 0.90)	
Fatigue		0.82 (0.74, 0.88)	0.70 (0.58, 0.80)
Sad or unhappy feelings	0.76 (0.66, 0.84)	0.80 (0.71, 0.87)	0.68 (0.55, 0.78)
Insomnia		0.69 (0.56, 0.79)	
Loose stools	0.77 (0.67, 0.85)		
Mouth or throat sores		0.90 (0.85, 0.94)	0.84 (0.77, 0.89)
Nausea	0.74 (0.63, 0.83)	0.78 (0.69, 0.85)	
Numbness/tingling in your hands or feet		0.88 (0.82, 0.92)	0.75 (0.63, 0.83)
Pain	0.87 (0.81, 0.92)	0.78 (0.69, 0.85)	0.86 (0.80, 0.91)
Shortness of breath		0.85 (0.77, 0.90)	0.75 (0.64, 0.83)
Vomiting	0.50 (0.32, 0.64)	0.36 (0.17, 0.53)	
Day 20 and day 21 (24-hour recall) (*N* = 77)			
Anxiety	0.79 (0.68, 0.86)	0.83 (0.75, 0.89)	0.79 (0.69, 0.86)
Constipation		0.64 (0.49, 0.76)	
Decreased appetite		0.92 (0.88, 0.95)	0.86 (0.79, 0.91)
Dry mouth		0.90 (0.84, 0.93)	
Fatigue		0.78 (0.68, 0.86)	0.87 (0.80, 0.91)
Sad or unhappy feelings	0.84 (0.77, 0.90)	0.85 (0.78, 0.91)	0.88 (0.82, 0.92)
Insomnia		0.66 (0.51, 0.77)	
Loose stools	0.55 (0.38, 0.69)		
Mouth or throat sores		0.97 (0.95, 0.98)	0.89 (0.84, 0.93)
Nausea	0.55 (0.38, 0.69)	0.72 (0.59, 0.82)	
Numbness/tingling in your hands or feet		0.92 (0.88, 0.95)	0.91 (0.86, 0.94)
Pain	0.88 (0.82, 0.92)	0.83 (0.75, 0.89)	0.83 (0.75, 0.89)
Shortness of breath		0.90 (0.85, 0.94)	0.88 (0.82, 0.92)
Vomiting	0.54 (0.35, 0.68)	0.51 (0.33, 0.66)	

Items with ICCs lower than 0.70 on days 6 and 7 tended to have high CVI values from days 0 through 6. For example, anxiety (I) had a CVI of 1.52, constipation (S) 1.43, sadness (I) with 1.43, insomnia (I) 1.06, vomiting (F) 2.00, and vomiting (S) 2.24. Greater intraindividual variability in symptoms measured by the items with lower test–retest reliability suggests that patients tend to change in their experience of these symptoms from day to day.

### Convergent and discriminant validity

Ten of the 14 symptomatic AEs included in this study are measured by more than one attribute (i.e., anxiety, sadness, fatigue, pain, nausea, vomiting, appetite, mouth sore, shortness of breath, and numbness). The bivariate correlations between the attributes for each of these 10 AEs ranged from 0.61 to 1.00 (median correlation, 0.75). The perfect bivariate correlation was between frequency and severity of nausea. Note that due to the skip pattern, the correlations may be higher compared to the situation where we do not implement the skip pattern.

**Fig. 2:**
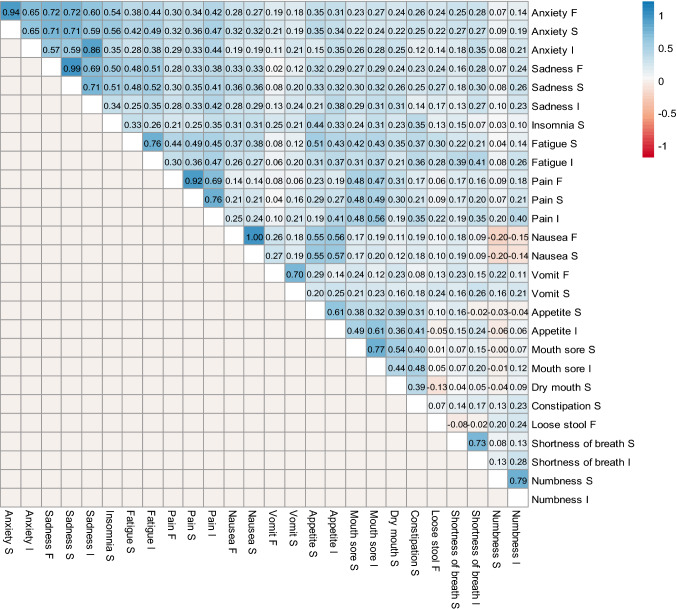
Heatmap of the bivariate correlations among PRO-CTCAE-24 h items on day 7. The variables that end with “F” indicate frequency, “S” severity, and “I” interference with daily activities

The bivariate correlations between items belonging to two conceptually related AEs were moderate to high (Fig. 2). For example, the items for anxiety and sadness were correlated at 0.57–0.86. Items for fatigue, pain, and insomnia were correlated at 0.21–0.49. Items for dry mouth and mouth sores were moderately correlated at 0.40–0.54. Other related domains that showed fair to low bivariate correlations included pain and mouth sores (0.47–0.56), nausea and appetite loss (0.55–0.57), and appetite loss and constipation (0.31–0.41). Symptoms that were less conceptually or mechanistically similar correlated at a much lower level. For example, PRO-CTCAE-24 h items measuring mouth sores were correlated at 0.01–0.05 with the item measuring diarrhea.

**Fig. 3:**
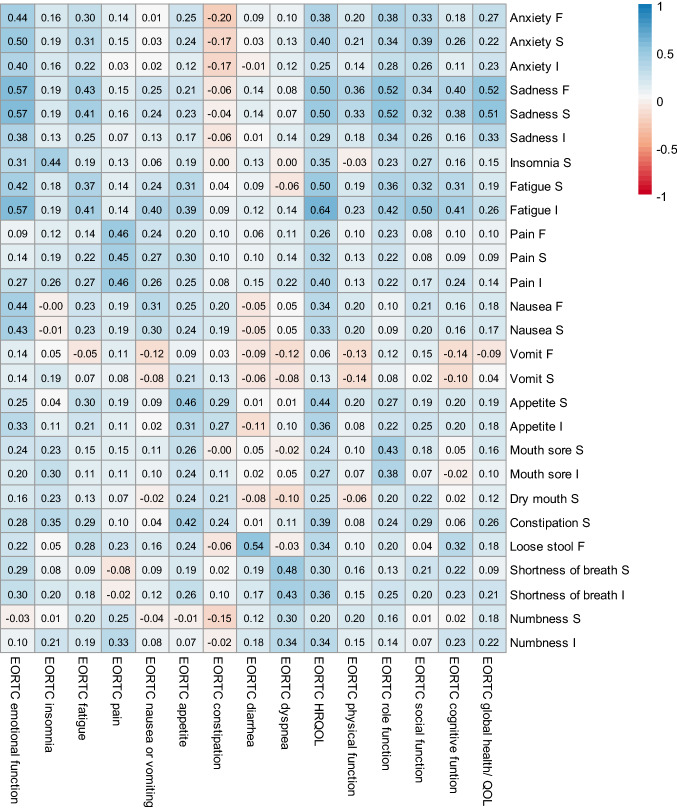
Heatmap of the correlations between the PRO-CTCAE-24 h on day 7 and EORTC QLQ-C30 scores at week 1. The variables that end with “F” indicate frequency, “S” severity, and “I” interference with daily activities

PRO-CTCAE-24 h items captured on day 7 were modestly correlated with conceptually relevant EORTC QLQ-C30 domains captured in week 1 in the expected directions (Fig. 3). For example, the QLQ-C30 emotional function scale was correlated at 0.38–0.57 with PRO-CTCAE anxiety (F, S, I) and sadness (F, S, I) items. Similarly, the QLQ-C30 insomnia scale was correlated at 0.44 with the PRO-CTCAE insomnia severity item. There were 26 PRO-CTCAE symptomatic adverse events where PRO-CTCAE-24 h correlated at 0.10 or above with the QLQ-C30 HRQOL (median *ρ* = 0.37); 23 PRO-CTCAE-24 h items were correlated at 0.10 or above with global health, 25 items with role function, 20 items with social function, and 19 items with cognitive function summary scores. PRO-CTCAE-24 h fatigue interference item demonstrated the strongest correlation with HRQOL (Spearman *ρ* = 0.64). Note that PRO-CTCAE-7d items had slightly higher correlations with the EORTC scales (average of all correlations in Fig. 3: 0.26) compared to the correlations between PRO-CTCAE-24 h items and EORTC scales (average of all correlations: 0.22) due to the matching recall period (Supplemental Figure).

### Responsiveness to change

#### PRO-CTCAE-24h with the PRO-CTCAE-7d measured from week 0 to week 1 as an anchor

**Fig. 4:**
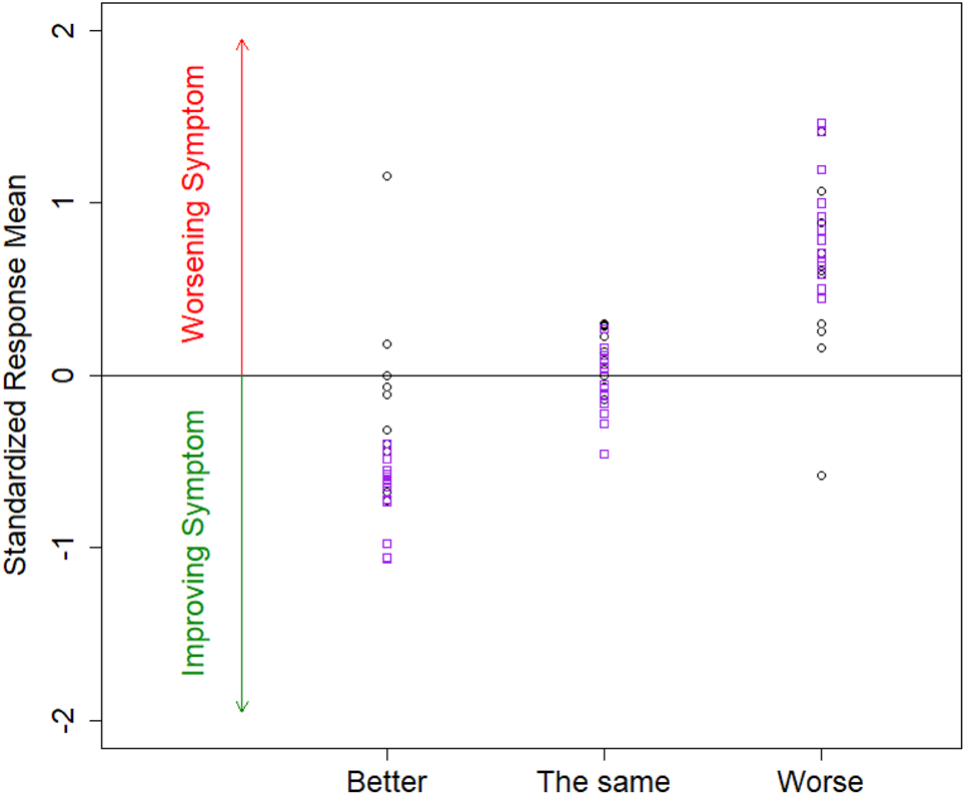
Standardized response means (SRMs) across 27 PRO-CTCAE-24 h items by the change category informed by PRO-CTCAE-7d items. The 15 SRMs in purple squares indicate items, in which changing trend of PRO-CTCAE-7d statistically significantly corresponded to the changing trend of PRO-CTCAE-24 h (*p* < 0.05 in Jonckheere-Terpstra test)

In this analysis of responsiveness using the one-sided Jonckheere-Terpstra, statistically significant (*p* < 0.05) monotonically decreasing PRO-CTCAE-24 h change scores (from day 0 to 7) were observed for 15 of 27 items (*p* < 0.001 for 6 items; sadness (F), loss of appetite (S), dry mouth (S), insomnia (S), mouth sores (S), and fatigue (S)). The median (range) SRM in patients in the improved category was − 0.52 (− 1.06, 1.15), whereas that in patients in the no change category was 0.04 (− 0.46, 0.30) and the worsened category was 0.71 (0.59, 0.92) (Fig. 4). In addition, statistically significant correlations (*p* < 0.05) were observed between change in PRO-CTCAE-24 h and change in PRO-CTCAE-7d for 19 of 27 items (*p* < 0.001 for 11 items): The median (range) for the correlation *ρ* was 0.32 (0.06 to 0.63).

### EORTC QLQ-C30 as an anchor

Statistically significant correlations (*p* < 0.05) were observed between PRO-CTCAE-24 h change scores and the changes in corresponding EORTC QLQ-C30 scales in 11 of 15 comparisons (*p* < 0.001 for 4 comparisons): The median (range) *ρ* was 0.34 (0.15 to 0.50).

## Discussion

To our knowledge, this is the first study investigating the validity and reliability of daily PRO-CTCAE reporting using a 24-hour recall period. We used PRO-CTCAE data with a 7-day recall period as the frame of reference for understanding the measurement properties of PRO-CTCAE responses captured daily using a 24-hour recall. Overall, the measurement properties of the 24-hour recall period were comparable to those previously reported for the 7-day recall period [5]. For example, the test–retest reliability values in the current study were comparable to those using a 7-day recall (test–retest reliability of 0.70 or greater was observed for 73% of the items with a 7-day recall period, and 77% using a 24-hour recall) [5]. Similarly, with both the 7-day and the 24-hour recall period, correlations between PRO-CTCAE items and conceptually related QLQ-C30 domains were stronger than those observed between PRO-CTCAE items and conceptually unrelated QLQ-C30 domains. CVIs for the PRO-CTCAE-24 h daily reports demonstrated considerable within-individual variability, particularly for mood changes, GI symptoms, and insomnia, underscoring the importance of capturing daily assessments when the shorter 24-hour recall period is employed.

The magnitude of the correlations between the change in PRO-CTCAE-24 h scores from days 0 to 7 and the change in EORTC QLQ-C30 scores from week 0 to 1 ranged from 0.15 to 0.50 (median 0.34), comparable to that observed using the PRO-CTCAE-7d change scores from week 0 to 1 (0.10 to 0.56 (median 0.43)) [5]

The median SRM in patients reporting improvement in the current responsiveness analysis was − 0.52 and worsening 0.71, compared to − 0.14 and 0.19 in the prior study investigating PRO-CTCAE-7d. Of note, the median SRMs are not directly comparable between the two studies: The criterion used for the SRM in the previous study validating PRO-CTCAE-7d was patients’ global impression of change in physical or emotional state. In addition, patients were asked to evaluate the change in physical or emotional state since the first time they answered the questionnaire, which could vary from one to six weeks ago. The criterion used in the current study was the change category informed by the change in PRO-CTCAE-7d from week 0 to 1.

Construct validity was supported by the modest associations observed among conceptually related PRO-CTCAE-24 h items. PRO-CTCAE-24 h items also had moderate associations with conceptually related EORTC QLQ-C30 symptom domains. Small–to-moderate associations between PRO-CTCAE-24 h items and the EORTC QLQ-C30 role function subscale were also observed. Except for two ‘vomiting’ items that had low prevalence, all items had at least fair-to-moderate relationships with overall HRQOL as measured by EORTC QLQ-C30. As shown by the analyses of responsiveness to change, PRO-CTCAE-24 h change scores from day 0 to 7 corresponded to the change categories informed by PRO-CTCAE-7d captured at week 0 and 1.

Strengths of this secondary analysis include prospective data capture in patients receiving cancer-directed therapy across multiple practice sites, inclusion of daily and weekly PRO-CTCAE assessments, and concurrent administration of the QLQ-C30. Levels of missing data were also relatively low, particularly in the first three weeks of data collection, especially considering that symptom assessments were administered daily. While IVR was used to administer PRO-CTCAE-24 h, web-based data capture for PRO-CTCAE-7d, and paper-based format for the EORTC QLQ-C30, a previous study [17] has demonstrated PRO-CTCAE mode equivalence.

There are several limitations to this study. We had small sample size and tested only a subset of all the PRO-CTCAE symptom terms. We did not investigate known group validity, due to a limited sample with impaired Eastern Cooperative Oncology Group performance scores (ECOG PS ≥ 2). Additionally, several AEs such as vomiting were uncommon in this study sample and received low endorsement. However, we assessed reliability and validity in 27 items that are common in cancer patients based on prior clinical trials [18]. Future research is needed to replicate and extend these findings using a larger sample and testing a larger subset of the 78 PRO-CTCAE symptom terms. For example, including relevant items that were not evaluated in the current study in future clinical trials as an exploratory endpoint can complement the findings. Most patients in the current study received daily treatment; a future study can broaden the patient population to those who do not receive daily treatment. We also note that a 24-hour recall may not be appropriate for all symptomatic AEs measured by PRO-CTCAE (e.g., sexual function) based on findings from other measurement systems [19].

These findings have practical significance for researchers investigating the tolerability of cancer treatments. Researchers may be particularly interested in using daily reporting to precisely characterize the onset and offset of symptomatic adverse events. For example, consider the case of surgeons who wish to compare patients’ symptom reports following surgery with and without intraoperative intraperitoneal chemotherapy. Administration of PRO-CTCAE-7d starting on post-op day 2, for example, is problematic because the recall period requires the respondent to report on their experience both before and after the surgical procedure. Pre-operatively a patient may have been entirely asymptomatic. In contexts where it is important to interpret a symptom constellation in relation to the treatment event (e.g., a sentinel treatment event such as surgery, initiation of a new systemic therapy or infusion of a cellular product), the use of daily reporting and a 24-hour recall, at least during the period surrounding the event of interest, can be especially valuable. At the same time, the administrative and analytic burden of daily reporting, and the potential for there to be more missing data must also be considered. There is also considerable within-patient variability across multiple assessments, which can make interpreting group-level data challenging. For example, for a symptom such as neuropathy which is expected to change over a longer timeframe (i.e., across multiple cycles of chemotherapy), daily assessments would not be an appropriate strategy. However, when the value of the information to be gained through daily assessment offsets these considerations, our analyses provide preliminary evidence to support study designs that employ daily PRO-CTCAE reporting using a 24-hour recall period. These findings also provide preliminary support for trial designs that accommodate two different recall periods, specifically a 24-hour recall for daily assessments surrounding an event under study, and reversion to the standard 7-day recall and weekly assessment intervals during the follow-up observation period. It is important to emphasize that recall periods and assessment schedules should be the same between study arms if between-arm comparisons are to be performed.

## Conclusion

Favorable measurement properties including test–retest reliability and responsiveness to change of PRO-CTCAE using a 24-hour recall has been demonstrated in a small sample of patients receiving active cancer treatment. The results of this study suggest that daily PRO-CTCAE reporting using a 24-hour recall can provide valid and reliable assessments that inform day-to-day variations in symptomatic adverse events and can be implemented in studies where the research aims warrant daily self-reporting of symptomatic adverse events.

## Supplementary Information

Below is the link to the electronic supplementary material.

Supplementary file1 (DOCX 127 kb)
