# Supplementary material for: Reliability and validity of PRO-CTCAE® daily reporting with a 24-hour recall period
Source: Qual Life Res. 2023 Mar 10;32(7):2047–58. doi: 10.1007/s11136-023-03374-5 (PMC10241696; doi:10.1007/s11136-023-03374-5)

Supplemental Table 1. Example PRO-CTCAE items with 24-hour recall

- In the last 24 hours, how OFTEN did you have loose or watery stools (diarrhea)?
- In the last 24 hours, what was the SEVERITY of your fatigue, tiredness, or lack of energy at its WORST?
- In the last 24 hours, how much did decreased appetite INTERFERE with your usual or daily activities?

Supplemental Table 2. Coefficient of variation for 27 prespecified PRO-CTCAE items during two assessment periods (Days 0 through 6, and days 15 through 21)

| **PRO-CTCAE Symptomatic AE Term** | **Coefficient of variation (median and inter-quartile range within individuals)** | | |
| --- | --- | --- | --- |
|  | **Frequency** | **Severity** | **Interference** |
|  |  |  |  |
| Days 0 through 6 (24-hour recall) |  |  |  |
| Anxiety | 0.91 (0.39, 1.55) | 0.94 (0.37, 1.71) | 1.52 (0.57, 2.24) |
| Constipation |  | 1.43 (0.68, 2.40) |  |
| Decreased appetite |  | 0.78 (0.37, 1.53) | 1.10 (0.56, 2.24) |
| Dry mouth |  | 0.37 (0.15, 0.96) |  |
| Fatigue |  | 0.38 (0.31, 0.77) | 0.71 (0.37, 1.46) |
| Sad or unhappy feelings | 1.06 (0.40, 1.92) | 1.10 (0.42, 1.71) | 1.43 (0.58, 2.45) |
| Insomnia |  | 1.06 (0.43, 1.67) |  |
| Loose stools | 1.76 (1.32, 2.45) |  |  |
| Mouth or throat sores |  | 0.74 (0.38, 1.59) | 0.94 (0.46, 2.45) |
| Nausea | 1.18 (0.63, 1.92) | 1.10 (0.65, 2.12) |  |
| Numbness/tingling in your hands or feet |  | 0.41 (0.00, 0.96) | 0.94 (0.55, 1.37) |
| Pain | 0.52 (0.24, 1.34) | 0.57 (0.26, 1.38) | 0.77 (0.43, 1.71) |
| Shortness of breath |  | 0.79 (0.43, 2.10) | 0.82 (0.38, 1.63) |
| Vomiting | 2.00 (1.46, 2.45) | 2.24 (1.55, 2.45) |  |
|  |  |  |  |
| Days 15 through 21 (24-hour recall) |  |  |  |
| Anxiety | 0.50 (0.23, 1.01) | 0.40 (0.30, 0.95) | 0.67 (0.37, 1.39) |
| Constipation |  | 1.06 (0.56, 1.71) |  |
| Decreased appetite |  | 0.32 (0.20, 0.90) | 0.56 (0.30, 1.71) |
| Dry mouth |  | 0.23 (0.00, 0.52) |  |
| Fatigue |  | 0.37 (0.19, 0.68) | 0.40 (0.22, 1.10) |
| Sad or unhappy feelings | 0.72 (0.35, 1.59) | 0.82 (0.37, 1.49) | 1.15 (0.41, 2.00) |
| Insomnia |  | 0.68 (0.37, 1.39) |  |
| Loose stools | 1.37 (0.71, 2.45) |  |  |
| Mouth or throat sores |  | 0.23 (0.00, 0.71) | 0.43 (0.22, 0.92) |
| Nausea | 0.70 (0.40, 1.25) | 0.85 (0.40, 1.42) |  |
| Numbness/tingling in your hands or feet |  | 0.39 (0.20, 1.03) | 0.58 (0.23, 0.87) |
| Pain | 0.35 (0.14, 0.60) | 0.31 (0.13, 0.66) | 0.47 (0.28, 0.99) |
| Shortness of breath |  | 0.39 (0.24, 1.26) | 0.54 (0.34, 1.15) |
| Vomiting | 2.00 (1.16, 2.45) | 1.71 (1.17, 2.06) |  |

Supplemental Figure. Heatmap of the correlations between the PRO-CTCAE-7d at week 1 and EORTC QLQ C30 scores at week 1


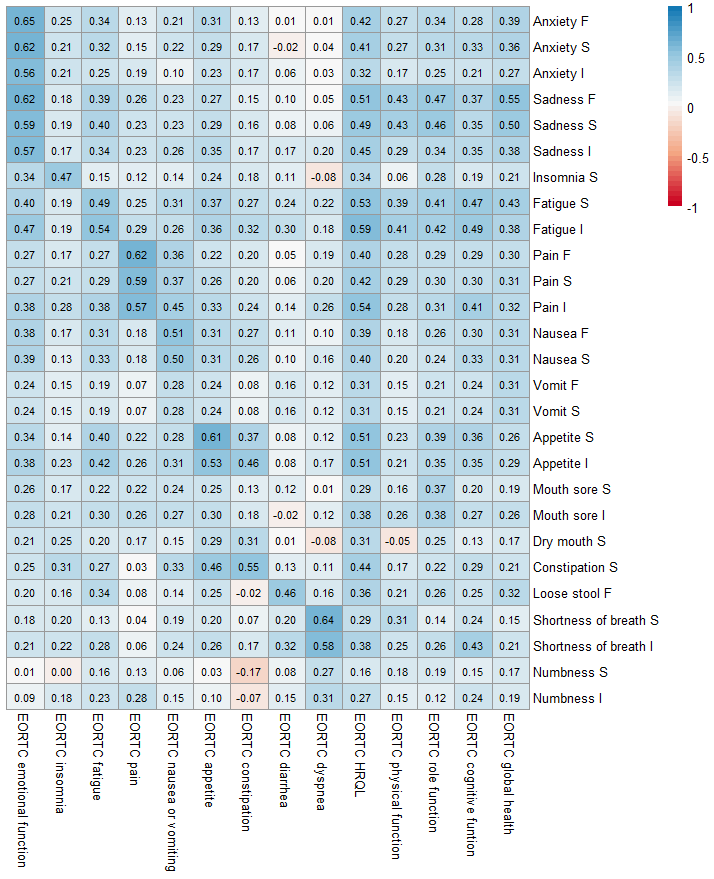

Supplement: Supplementary file 1 — Supplementary file1 (DOCX 127 kb) [file 11136_2023_3374_MOESM1_ESM.docx]
